# Knowledge, Attitude and Practice of selected *Dinacarya* practices among BAMS Students in Kerala - A Cross Sectional Study

**DOI:** 10.1016/j.jaim.2026.101320

**Published:** 2026-03-11

**Authors:** K. Aakash, Anupama Krishnan

**Affiliations:** a Department of Swasthavritta Evam Yoga, Global Institute of Ayurveda, Rajkot, Gujarat, India; b Department of Swasthavritta Evam Yoga, Vaidyaratnam P.S Varier Ayurveda College, Kottakkal, Malappuram, Kerala, India; c Affiliated to Kerala University of Health Sciences (KUHS), Thrissur, Kerala, India

## Abstract

**Background:**

Ayurveda students in India learn *Dinacarya* practices (daily routines) during BAMS course (Undergraduate Ayurveda medical education course), with limited research on its awareness and adoption. A valid database on Knowledge-Attitude Practice (KAP) status of *Dinacarya* practices is ideal for self-evaluation and further student improvement.

**Objective:**

To assess the KAP status of selected *Dinacarya* practices among BAMS students in Kerala.

**Materials and Methods:**

Cross-sectional study among 216 students aged 18-27 years, using stratified two-stage sampling from two strata - Government/Aided and Private Ayurveda colleges in Kerala (7 colleges). Semi constructed KAP questionnaire on selected ten *Dinacarya* developed through literature review, focus group discussion (FGD),content and face validity checking. Population estimates calculated using survey weights and Multiple linear regression (MLR) to identify predictors of KAP components and composite scores was used.

**Result:**

Population-weighted mean K, A, P and total KAP scores of Dinacarya came to 6.65 (SE=0.48), 3.25 (SE=0.22), -5.01 (SE=0.29) and 4.89 (SE=0.61) respectively. MLR analysis revealed that knowledge was primarily determined by institutional factors, attitude reflects lifestyle pathways and Practice projects multilayered predictors encompassing environmental and institutional factors.

**Conclusion:**

Weighted KAP analysis revealed knowledge scores exceeding attitude and practice scores, indicating a knowledge-practice gap. Institutional entity emerged as overall determinant, residence type and psychological disposition are crucial factors.Health behaviors practice progressively decline with advancement in academic years.

## 1 Introduction

Healthy habits/behaviors among medical students claim prime importance, as they are future physicians and health educators [1]. Theory of planned behavior designates attitudes, behavioral intentions, subjective norms social norms, perceived power and perceived behavioral control as six factors influencing human and community behavior [2]. Student behaviors are reflected as temporary; nevertheless, imbibed unhealthy habits generally persist in adult life [3]. Health care practices pursued by medical students and physicians motivates populations to embrace healthy lifestyles [1]. *Dinacharya* - An Ayurvedic health enhancement measure, is daily regimen designed to maintain the ideal lifestyle, which optimally connects to the circadian rhythms and rhythm of nature [4]. In India, Ayurveda college students learn *Dinacharya* during BAMS course, wherein, self-practice of *Sila Saucachara* (habits maintaining cleanliness/purity in thoughts and acts) and *Swasthavṛitta Vihita Karma* (actions favoring individual's health) by students and physicians is recommended [[5], [6], [7]]. *Adhiti* is the initial stage of knowledge acquisition and assimilation, followed by *Bodha* which involves comprehending and internalizing. The next stage is *Acharana,* which equates to application and practice. After completion, one is expected to be capable of *Pracharana,* which entails preaching, teaching, advocacy, and knowledge distribution [8].

However, there are no studies substantiating the extent of knowledge, attitude and practice regarding *Dinacharya* among BAMS students. Hence, this cross-sectional study to assess KAP Status of *Dinacharya* practices among BAMS students in Kerala (as primary objective) was planned and conducted using a validated questionnaire (scale development and validation was a secondary methodological contribution of this study).

## 2 Materials and methods

### 2.1 Study design

Cross-sectional study utilizing stratified two-stage cluster sampling (2- stages - (1) Selection of Colleges; (2) Selection of Participants from Colleges).

### 2.2 Study setting

Selected 7 Ayurveda Colleges in Kerala during the period of 2022 February - 2023 July (18 Months).

### 2.3 Participants

#### 2.3.1 Inclusion and exclusion criteria

BAMS students of Ayurveda colleges in Kerala of either sex coming under age group of 18–27 years, who were ready to give written consent to participate in the study, were included in study. The students from the college where researchers belong, the newly joined First year junior batch (2022 Admission batch) and House surgeons (2017 batch) were excluded in the study.

#### 2.3.2 Sampling frame

Sampling frame included students satisfying the inclusion criteria, from 18 Ayurvedic colleges across all 14 districts of Kerala. A stratified two-stage sampling design was applied beginning with randomly selecting colleges within each stratum, followed by randomly selecting students from selected colleges. Finally, two strata emerged based on the college category - Government/Aided (Stratum 1) (5 colleges) and Private (Stratum 2) (13 colleges).

#### 2.3.3 Sample size calculation

Sample size was ascertained for estimating population mean KAP scores with 95 % confidence and margin of error ≤1.0 point. Initial stratification considered 18 colleges across 14 districts. Pilot analysis showed minimal between-district variance in KAP scores. Final stratification by college type (Government/Aided vs Private) captured 85 % of systematic variance. The basic sample requirement for simple random sampling being 78 students, followed by adjustments for design effects due to clustering (Deff = 2.8) and stratification efficiency (factor = 0.85), a required sample of 187 students emerged. Nevertheless, actual sampling accomplished 216 students across seven colleges, exceeded required sample (n = 187) by 15.5 %, facilitating sufficient power for population estimation and stratum comparisons. Population-weighted estimates showed minimal design effects (−0.040 to +0.052), confirming efficient stratification.

#### 2.3.4 Sample selection

BAMS students (216) were recruited from seven randomly selected Ayurveda colleges by proportionate allocation:

• Stratum 1 (Government/Aided): 62 students from 2 colleges (out of 5 colleges)
• Stratum 2 (Private): 154 students from 5 colleges (out of 13 colleges)

#### 2.3.5 Survey weights

Sampling weights were designed as the inverse of selection probabilities:

Stage 1 probability: (colleges selected in stratum)/(total colleges in stratum)
Stage 2 probability: (students selected in college)/(total students in college)

Final weight = 1/(Stage 1 probability × Stage 2 probability).

### 2.4 Variables

• K, A, P and total KAP status of selected 10 *Dinacharya*
• Selected personal and demographic variables of participants.

### 2.5 Data sources/measurement

#### 2.5.1 Preparation of preliminary questionnaire

Literature sources included Ayurveda classical texts, journals, news reports and scientific articles published between 1990 and 2023, which were searched using the key words like *Dinacharya,* lifestyle, medical students, health education etc. Focus group discussion (FGD) with Ayurveda Physicians having more than three years of undergraduate teaching experience belonging to department of Swasthavritta, Samhita, Sanskrit and Siddhanta (selected through purposive sampling) was conducted for selecting and fixing ten *Dinacharya and* K-A-P question domains for each *Dinacharya* (Fig. S1).

The chosen *Dinacharya* were *Brahma Muhurtha Uttana* (early morning awakening), *Dantadhavan* (tooth brushing), *Anjana* (application of collyrium), *Nasya* (nasal instillation), *Gandusha* (Oil pulling), *Abhyanga* (oil massage), *Vyayama* (physical exercise), *Snana* (Bath), *Ahara* (food intake) and *Nidra* (sleep). The knowledge domain for each *Dinacharya* highlighted the knowledge about procedure, frequency, materials used, time of practice, specific characteristics and benefits. The attitude and practice domains encompassed the willingness/opinion/belief and practice/avoidance respectively (Table S1).

The KAP questionnaire was framed with the 30 question domains (one for each *Dinacharya*'s Knowledge, Attitude, and Practice) The case record form (CRF) furnished respondent's personal and demographic details. The validated KAP questionnaire (Doc.S1) along with CRF and Consent form was employed for data collection.

Face validity checking was ascertained among five post graduate students of the researcher's department. The content validity ascertained with the help of five physicians having more than ten years of clinical experience, emerged with content validity index (CVI) scores of 0.90. The questionnaire was tested for reliability using Cronbach's coefficient α. Correlation between the respective item and the total sum score (without the respective item) internal consistency of the scale (coefficient α) if the respective item would be deleted were checked. A score of 0.644, indicated equal importance of all questions and good internal consistency. Pilot study was deployed among 15 undergraduate students for cognitive reasoning of V.P.S.V. Ayurveda College, subsequently, the questionnaire was reframed based on the obtained feedback.

#### 2.5.2 Ethical clearance and concerns

The study was approved by Institutional ethics committee (IEC) prior to the starting of work (approval no: IRB/Doc/31/21 dated 26–07–2021). Furthermore, the questionnaire contained a copy of confidentiality agreement stating the purpose of the study and assuring strict confidentiality of the respondents. The respondents were asked to sign on consent from with a declaration stating that their participation in the study was purely voluntary and the responses given by them were based on their own individual perceptions and that they were not compelled to respond in any particular way by the investigators or by any other authority.

#### 2.5.3 Collection of data

Permission from the concerned head of the institutes of selected seven Ayurveda colleges were priorly procured (by in-person visit by the first author and online communication from the researcher's institution) to conduct survey in first, second, third and fourth-year BAMS classes. Along with distributing participant information sheet (PIP), the researcher verbally explained the content of the study's goal and the participant confidentiality. Thereafter, participant's doubts were cleared and their written consent were collected before the distribution of CRF and KAP Questionnaire. The participants were allotted a time of 30–45 min to complete self-administered questionnaires. The data was gathered as completed Case Record Forms (CRF) and KAP Questionnaire. Data cleaning and data entry of scored and unscored data were ensured on the same day of data collection. The scoring of K-A-P data was conducted by considering similarity and dissimilarity of their response with the classical Ayurveda principles regarding each of the *Dinacharya*. The responses closer to the ideal Ayurveda classical point of view were assigned higher positive scores and those more dissimilar/inappropriate, were awarded negative scores (Doc.S2).

### 2.6 Bias

The students from the college, where researchers belong, were excluded from study to avoid response bias. The newly joined first year junior batch (2022 Admission batch) and house surgeons (2017 batch) were excluded in the study, to avoid selection bias.

### 2.7 Statistical analysis

The collected data compiled using Microsoft Excel 2012 was used for working out different frequency tables, cross tabs and descriptive statistics of the concerned variables.

**Weighted Population Estimates:** Population means, totals, and confidence intervals were calculated using survey weights in a two-stage stratified design with R survey package. The survey design encompassed - Primary sampling units (colleges) and secondary units (students), stratification by college type, sampling weights accounting for unequal selection probabilities and finite population corrections.

**Multivariable Analysis:** Predictors of Individual KAP components (K Total, A Total, P Total) and Composite KAP scores (KAP Grand Total) were identified by Multiple linear regression (MLR) with forward stepwise selection. MLR analysis was performed using R statistical software (version 4.4.2 (2024-"Pile of Leaves") with forward stepwise selection based on Akaike Information Criterion (AIC). All MLR models underwent - Normality testing of residuals, Homoscedasticity assessment, Multi collinearity evaluation (VIF<2.5) and Outlier removal (1 case excluded, final n = 215).

Twenty-two potential predictors were evaluated including demographic (age, gender), institutional (college stratum, year of study), lifestyle (residence type, diet patterns, sleep habits), and health-related factors (psychological disposition, bowel habits). Variable selection used AIC with comprehensive model diagnostics including normality testing, homoscedasticity assessment, and multi collinearity evaluation (VIF <2.5).

**Significance Testing:** Statistical significance was set at p < 0.05. Attempt to account for the complex survey design using appropriate weights was made, however, the stepwise regression hinted problems of convergence and hence, un weighted analysis was resorted to at this step. This minor difference is not likely to sabotage the results much; except in terms of differences in p values of individual coefficients.

**Missing Data:** Missing data were assessed at the level of questionnaire completion. A total of 226 participants were approached, of which 216 provided complete responses, giving a non-response rate of 10/225 = 4.4 % and a response rate of 95.6 %. To maintain the target sample size, the questionnaire was reissued to additional eligible participants until the required 216 complete responses were obtained. Thus, the final analytic dataset contained no missing cases at the participant level. Item-level missing within completed questionnaires was negligible.

## 3 Results

### 3.1 Descriptive data

Among 216 participants (final analytical sample: 215 after outlier elimination), population-weighted characteristics included mean age of 21.71 ± 1.422 years, with 74.5 % females. The study finding revealed that the majority belonged to Private Ayurveda colleges (72.3 %), were hostellers (78.2 %) and did not have Ayurveda family background (81.9 %). Most prevalent *Prakriti* (Body Constitution) (self-assessed) was *Vata-Pitta Prakriti* (32.9 %) and *Madhyama sattva* (Moderate mental strength) (79.2 %) (Table S2).

### 3.2 Outcome data

Table 1 shows prevalence of different responses on Knowledge, Attitude and Practice among each of the ten selected *Dinacharya* (K, A & P Status) [also refer Table S1& Doc.S1].

**Table 1:** Prevalence of knowledge, attitude and practice on *Dinacarya* (K, A& P status).

Sl. No	K Domain	Unknown	Known	A Domain	Strongly Agree/Agree	Strongly Disagree /Disagree	Don't Know	P Domain	Always/Frequently	Occas- ionally	Rarely/Never
1	K1	71.8 %	28.2 %	A1	76.4 %	14.4 %	9.3 %	P1	14.4 %	20.8 %	64.8 %
2	K2	11.1 %	88.9 %	A2	91.7 %	6.0 %	2.3 %	P2	85.6 %	5.6 %	8.8 %
3	K3	24.1 %	75.9 %	A3	47.7 %	40.7 %	11.6 %	P3	21.8 %	20.4 %	57.9 %
4	K4	20.4 %	79.6 %	A4	89.4 %	4.2 %	6.5 %	P4	4.2 %	6.5 %	89.4 %
5	K5	50.0 %	50.0 %	A5	88.9 %	6.5 %	4.6 %	P5	6.0 %	13.0 %	81.0 %
6	K6	39.8 %	60.2 %	A6	84.7 %	9.7 %	5.6 %	P6	34.3 %	37.5 %	28.2 %
7	K7	78.2 %	21.8 %	A7	81.0 %	13.0 %	6.0 %	P7	23.6 %	16.7 %	59.7 %
8	K8	37.0 %	63.0 %	A8	81.0 %	9.7 %	9.3 %	P8	8.8 %	24.1 %	67.1 %
9	K9	44.9 %	55.1 %	A9	95.4 %	4.2 %	0.5 %	P9	46.8 %	22.7 %	30.6 %
10	K10	11.1 %	88.9 %	A10	68.5 %	21.8 %	9.7 %	P10	68.5 %	16.7 %	14.8 %

### 3.3 Main results

The individual mean K, A, P and KAP scores (Attained by adding individual K, A & P scores - represent overall approach towards *Dinacharya*) for each *Dinacharya* are presented in Table 2. The Positive/correct knowledge about *Dinacharya* was most visible in the case of *Nidra* (K = +1.58) and least visible in the case of *Brahma Muhurtha Uttana* (K = −0.74). The Positive Attitude towards *Dinacharya* surfaced in *Ahara/Bhojan* (Food intake) (A = +1.39) and least in *Abhyanga* (Oil massage) (A = −1.07). Proper Practice of *Dinacharya* was reported in *Snana* (P = +1.06) and least in *Nasya* (Nasal instillation) and *Dantadhavan* (Tooth brushing) (P = −1.50). Positive overall approach (Total KAP Status) towards *Dinacharya* was revealed in *Anjana* (Application of collyrium) (KAP = +1.76) and least elicited in *Vyayama* (Physical Exercise) (KAP = −1.75).

**Table 2:** Individual mean K-A-P scores and Total KAP scores of *Dinacarya*.

Sl.No	Dinacarya	K (−2 to +2)	A (−2 to +2)	P (−2 to +2)	KAP (−6 to +6)
1	*Brahmamuhurta utthāna*	−0.7407	+0.8333	−0.7130	−0.6204
2	*Dhantadhavana*	+1.5556	+1.1481	−1.5046	+1.1991
3	*Anjana*	+1.2222	−0.0787	+0.6250	+1.7685
4	*Nasya*	+1.3981	+1.2593	−1.5046	+1.1528
5	*Gandusa*	+0.0370	+1.1204	−1.3333	−0.1759
6	*Abhyanga*	+0.4167	−1.0787	+0.1250	−0.537
7	*Vyayama*	−0.1667	−1.0463	−0.5370	−1.75
8	*Snana*	+0.8889	−1.0602	+1.0602	+0.8889
9	*Ahara*	+0.4537	+1.3935	−0.2546	+1.5926
10	*Nidrà*	+1.5833	+0.7639	−0.9769	+1.3703
	Total Score	+6.6481	+3.2546	−5.0139	+4.8888

Population Estimates for KAP Components are presented in Table 3. The Mean K, A, P and Total KAP scores of *Dinacharya* among 216 BAMS students are +6.65, +3.25, −5.01 and + 4.89 respectively. The population-weighted analysis revealed minimal design effects, indicating that the stratified sampling provided efficient estimates. However, the weighted estimates provide the appropriate population-level inferences.

**Table 3:** Population estimates for KAP components.

Sl.No	Component	Population Mean (SE)	95 % CI	Sample Mean	Design Effect^a^
1	Knowledge Total	+6.65 (0.48)	[5.70, 7.60]	+6.61	+0.04
2	Attitude Total	+3.25 (0.22)	[2.82, 3.68]	+3.21	+0.04
3	Practice Total	−5.01 (0.29)	[-5.58, −4.44]	−5.05	+0.04
4	KAP Grand Total	+4.89 (0.61)	[3.69, 6.09]	+4.77	+0.12

a Design effect = Population estimates - Sample mean.

### 3.4 Other analyses

#### 3.4.1 Multiple linear regression analysis

##### 3.4.1.1 Knowledge component (R^2^ = 7.7 %, F = 4.387, p = 0.002)

The knowledge model identified four predictors:

College stratum (Private vs Government/Aided): β = +2.69, p = 0.010 (Only statistically Significant predictor), Socioeconomic status: β = −2.12, p = 0.083 (Marginally significant, counter intuitive negative association), Sleep type: β = −2.00, p = 0.092 (Marginally significant) & Ayurveda family history: β = −2.00, p = 0.099 (Marginally significant). Dominance of institutional factors hints that curriculum rigor and teaching traits create differential learning outcomes that may supersede individual characteristics.

##### 3.4.1.2 Attitude component (R^2^ = 7.1 %, F = 3.205, p = 0.008)

Attitude model focusses on four significant predictors:

Diet type (Vegetarian Diet): β = −1.55, p = 0.033, Psychological temperament (*Avara sattva* - Low mental strength): β = −2.29, p = 0.018, Year of study (3rd/4th year Students): β = −0.94, p = 0.032 & Daytime sleep duration: β = +0.70, p = 0.037. The pattern reveals attitudes being swayed by personal behaviours and experiences; with formal education being less influential.

##### 3.4.1.3 Practice component (R^2^ = 16.2 %, F = 4.411, p < 0.001)

The practice model showcased seven predictors:

Residence type (Rent room): β = −2.58, p = 0.001 (strongest predictor),College stratum: β = +2.20, p = 0.001,Year of study (3rd/4th year Students): β = −1.34, p = 0.019, Psychological disposition (*Avara sattva*): β = −2.65, p = 0.037, Socioeconomic status: β = +1.37, p = 0.061 (Marginally significant), Diet type: β = +1.97, p = 0.054 (Marginally significant) & Ayurveda family history: β = +1.11, p = 0.160 (non-significant but retained). The complex predictor list reveals plural barriers that must align for purposeful behaviour enactment.

##### 3.4.1.4 Composite KAP analysis (R^2^ = 13.3 %, F = 3.957, p < 0.001)

Six predictors emerged in the composite model namely, College stratum: β = +4.16, p = 0.002 (Strongest predictor), Bowel frequency (2 Times/Day): β = −2.84, p = 0.022, Residence type (Rent room): β = −3.28, p = 0.044, Age: β = −1.21, p = 0.034, Sleep type: β = −2.27, p = 0.135 (non-significant but retained), Year of study: β = +2.41, p = 0.159 (non-significant but retained).

Table .4 shows Summary of MLR Results by K-A-P Components.

**Table 4:** Summary of MLR results by KAP component.

Sl.No	Component	Primary Pathway	Key Predictors	R^2^	Interpretation
1	Knowledge	Institutional	College stratum (β = +2.69∗∗∗)	7.7 %	Educational quality determines knowledge acquisition
2	Attitude	Lifestyle	Diet, psychology, sleep	7.1 %	Personal experiences frame attitudes
3	Practice	Multi-layered	Environmental + institutional + individual	16.2 %	Multiple barriers impact execution
4	Combined KAP	Institutional + Health	College quality + physiological indicators	13.3 %	Institutional entity influence performance

∗∗∗p < 0.05, ∗∗p < 0.01, ∗∗∗p < 0.001.

## 4 Discussion

### 4.1 Interpretation

The standards, motives, challenges and outcome of Ayurveda education have been a debate initiating and attention drawing area, during past years [9]. The present study tried to assess KAP Status of *Dinacharya* practices among BAMS students: since, KAP studies are desirable for grounding of regulations, upholding apt practices, and scheming resourceful initiatives [10]. Also, mediated behavioral change is an established method for successful and effective learning transfer, which would collectively serve as a skill booster [11].

This study presents the first KAP assessment of *Dinacharya* practices using stratified two-stage sampling with survey weights, inducing valid population-level estimates rather than sample-specific findings. The attributed design effects were negligible (range: 0.040 to +0.052), signifying effectual stratification, meanwhile generalizability to the target population (BAMS students in Kerala) is assured by the weighted estimates. Multiple linear regression projects component-specific predictor patterns, and the KAP components pursued different causal pathways, challenging linear progression.

In this study, the K-A-P Score evaluation reveals high prevalence of appropriate knowledge, improper practice, moderate positive attitude and overall positive approach on *Dinacharya* among students. In the present study, population-weighted estimates endorse a notable Knowledge-Practice gap, with knowledge scores (+6.65, SE = 0.48) overtaking practice scores (−5.01, SE = 0.29). This 11.66-point difference in educated population highlights a genuine implementation gap, demanding systems-level mediations, far more than traditional knowledge-focused education. The significant negative practice scores, declare weak health behaviors, in spite of educational exposure, implying that knowledge procurement alone does not ensure behavior change.

The variable ‘strata of college’ emerged as the statistically most influencing factor, significantly associated with Knowledge, Practice and overall KAP status. In the present study, Private Ayurveda colleges students secured higher scores in Knowledge, Practice and KAP of *Dinacharya*, when compared to Government/Aided Ayurveda colleges, propelling to consider variables outweighing individual student characteristics. Conversely, cross-sectional study by Chellaiyan VG et al., hints towards better academic performance and perception in Govt medical institutes compared to their private counterparts [12].

The present study reveals that the Attitudes pattern reflects lifestyle pathway dictated by personal behavioral patterns, framed by lived experiences, which overrule formal educational involvement. Also, the Practice domain showcases a multi-layered pathway conjugating individual, institutional and environmental factors simultaneously. The seven-predictor model of the practice domain, endows the behavior change complexity.

Interestingly, residence type emerged as significant factor influencing implementation capacity in the study. The participants residing in rent rooms exhibited negative approach in Practice and total score KAP than those who residing in house and hostel. This suggests the need for environmental interventions rather than individual-level initiation. Coherently, study conducted by Khan M Set al., among medical day scholars and hostellers, has highlighted the influence of residence environment on personality traits [13].

*Avara sattva* was found to be negatively associated with Attitude and Practice, denoting the influence of psychological disposition on health behavior [14]. Nevertheless, prominence in self-reported *Madhyama sattva* in the study's observation may be addressed, with an eye to central tendency bias [15].

The queer pattern showing higher bowel frequency (2times/day) linked with lower KAP scores, calls for further attention into the association between gastrointestinal awareness and health behavior.

Notably, the study results among Ayurveda students also resonates with derailed eating and disordered eating behaviors, reported among medical students by earlier researchers [16,17]. Nearly half of the participants were unaware about healthy eating habits explained in Ayurveda, which includes the time gap between subsequent food intake and features of proper digestion. However, good number of participants echoed a positive attitude towards regular timing and proper digestive power before food intake. Then again, the same positivity failed to reflect in practice domain. Mindful eating habits were also not traceable in nearly half of the participants and they indulged in frequent mobile use or exposed to television watching or other audio video usage during food intake.

Among the participants, one among ten were overweight or severely obese. Researches point towards disrupted eating pattern, food addiction, and higher body fat among medical students [17]. Also, high stress levels and living away from home, worsens dietary routine, which in turn, impacts both health and academic performance [18].

Majority of BAMS students abstained from exercise, which is consistent with the decline in exercise and sports among medical students [[19], [20], [21]]. Only a minority appraised proper knowledge of *Ardha Shakti Vyayama* (ideal endpoints of exercise) and practiced *Vyayama* aligned to *Ritu* (Season), *Sharira bala* (Body strength) and *Ahara*. Also, lack of proper physical education training facility/faculty was reported by many participants, which aligns with the reported barriers to exercise such as time constraint and unsuitable schedules [22]. Enhanced physical activity are positively linked with professional effectiveness and yoga classes are recommended in medical schools as stress negotiators [23,24]. Conversely, mean KAP score of *Vyayama* in the present study, indicated a negative approach among BAMS students, who have yoga training as a part of their curriculum. Additionally, significant prevalence of actual obese, overweight and underweight in medical students have been reported [25]. Among the study participants, a sizable portion revealed deviation from desirable BMI status, hinting towards distorted exercise and nutrition trend.

The promising effect of *Abhyanga* in reducing subjective stress experience is relevant, when the studies show one among three of the medical college students globally have anxiety and depression [[26], [27], [28]]. With more than one third of participants being unaware about the mandatory *Abhyanga* body sites and more than one among four of them, never or rarely practicing it, undermines the importance of simulation-based teaching methods in Ayurveda [29].

Systematic review by Michaelson V et al., highlights family as the most intimate unit in health context [30]. Agreeably, present study also noted the family influence on health promotion behavior, exceeding the academy influence. However, the Ayurveda family background does not emerge as statically significant.

More than half of the participants reported insufficient sleep: congruently disturbed sleep and day sleep summed up to a sizable proportion. Similar trends of sleep difficulties and undesirable sleep behaviors have been elicited among medical students [[31], [32], [33], [34], [35], [36]]. The time of class initiation in the morning can influence the student's sleep wake pattern [37]. An interesting observation of the study was daytime sleep being positively associated with attitude domain.

Future doctors have insufficient knowledge with more misconceptions regarding sleep, circadian rhythm and sleep disorder [38,39]. Almost three out of every four BAMS students were unaware of the time of *Brahma Muhurtha* (Early morning time ideal for awakening). Not withstanding, majority of students echoed a positive attitude towards waking up in *Brahma Muhurtha*: yet, only a staggering number were practicing it. Hence, a learning approach focusing on action is desirable for extended skill enhancement in real world experiences [40,41]. The harmful impact of sleep deprivation, deteriorating exercise and sleep habit among the participants needs to addressed.

Since optimum exercise is proven protective factor for sleep quality, the curriculum adjustments in medical schools are prerequisite for promoting sleep hygiene and health sleep behavior [36,42].

The least practice score in *Nasya* and *Dantadhavan* (P = −1.50) shows the reluctance of participants in following these *Dinacharya* practices in daily life. A positive overall approach towards *Anjana* surfaced (KAP = +1.76); yet, since the material used for *Anjana* was not addressed, there prevails an uncertainty regarding use of classically mentioned methods among the participants.

The ascending years of study of students had a negative association with Attitude and Practice. The irony of medical scholars showcasing obvious pattern of undesirable lifestyle habits and systematically swaying away from what they preach has been a much debated; yet unresolved terrain [43,44]. Researchers, educators and physicians have unanimously voiced the long-time pressing need of policy research in Ayurveda education for reframing the knowledge, skill, attitude and competency domains [8,45]. Our study also indicates towards existence of problems in developing and maintaining ideal standards on knowledge, attitude and practice of healthy lifestyle among BAMS students, in spite of their institutional training.

### 4.2 Limitations

The cross-sectional design does not provide causal inference. This KAP study was limited to ten *Dinacharya* and considered only one domain each for Knowledge, Attitude and Practice assessment for each *Dinacharya.* The probable difference between the Knowledge/Attitude/Practice status of different domains of a single *Dinacharya* itself, cannot be excluded. The relatively low variance explained (7.1–16.2 %) points towards substantial unaccounted influences, urging future exploration. The authors acknowledge the possibility of relevant cultural influence, though, it was not statistically detectable in the particular study. Furthermore, socio-economical or regional variation, triggering residual confounders cannot be ruled out. The logistics of visiting the geographically widely separated seven Ayurveda college across the state was a major challenge encountered during the study.

### 4.3 Generalizability & recommendations

Advanced and extended survey studies are recommended for eliciting specific factors influencing KAP Status of *Dinacharya*, along with ruling out residual confounders. The similar study can be conducted among other *Ayurveda* communities like PG/PGD scholars, teachers, medical officers, general registered practitioners and nurses. Thus, an overall KAP status of *Dinacharya* among persons who preach is worthwhile, since it would draw attention on the need of self-evaluation and self-correction in Ayurveda educational system and Ayurveda fraternity. Also, similar KAP studies can be conducted on epidemiologically relevant Swasthavritta concepts (*Ritucharya*, *Virudha Ahara, Sadvritta, Pathya - Apathya, Vega Dharana* etc.) and Ayurveda basic fundamental principles, public health endeavors and integration possibilities.

## 5 Conclusion

This study revealed significant gaps in KAP on *Dinacharya* among BAMS students in Kerala. The weighted KAP analysis revealed knowledge scores exceeding Attitude and Practice scores, indicating a Knowledge-Practice gap. KAP components project diverse causal pathways, necessitating customized mediation. Institutional entity emerges as crucial overall determinant, while physiological health indicators like bowel frequency surface as novel predictive factor. Living arrangements significantly influence carrying out health practice, suggesting need for environmental interventions rather than individual-level initiation. Health behaviors practice progressively decline with advancement in academic years, urging serious speculation. The influence of psychological disposition on health behavior also surfaced with *Avara sattva,* found to be negatively influencing attitude and practice domain.

Optimum institutional measures and tailored educational policy and self-motivation coupled with correction, are pivotal to ensure adherence to Ayurvedic lifestyle principles among its future flag bearers.

## Author contributions

AAK & ANK conceptualized and designed the survey study. AAK done the validation of KAP questionnaire and conducted survey study under the guidance and supervision of ANK. Both AAK & ANK drafted, reviewed and edited the manuscript for final submission.

## Declaration of generative AI in scientific writing

No generative AI tools were used in the preparation of this manuscript. All content was created and written by the authors.

## Funding sources

No funding was received for this work.

## Conflict of interest

The authors declare that they have no known competing financial interests or personal relationships that could have appeared to influence the work reported in this paper.
