# Supplementary material for: Knowledge, Attitude and Practice of selected Dinacarya practices among BAMS Students in Kerala - A Cross Sectional Study
Source: J Ayurveda Integr Med. 2026 Mar 11;17(2):101320. doi: 10.1016/j.jaim.2026.101320 (PMC12995569; doi:10.1016/j.jaim.2026.101320)
Supplement: Multimedia component 1 [file mmc1.pdf]

Fig S1 - **SOCIOGRAM OF FGD**

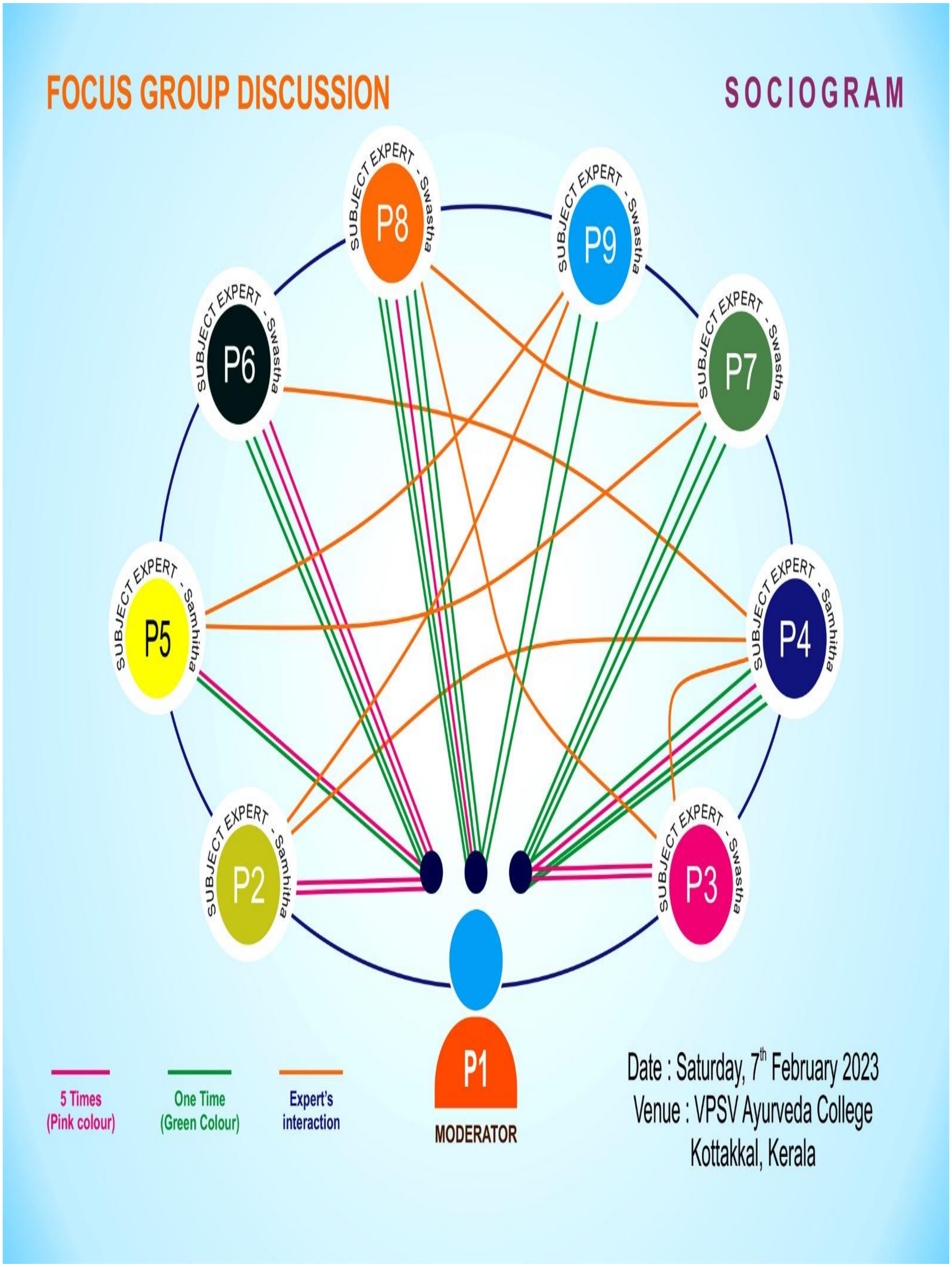

Tab S1- **KAP DOMAINS OF 10 SELECTED DINACARYA**

Table S1 – K-A-P Domains for selected Dinacarya

**DINACARYA**	**KNOWLEDGE DOMAINS (K)**	**ATTITUDE DOMAINS (A)**	**PRACTICE DOMAINS (P)**
Brahmamuhurtha uttana (Early morning awakening)	Time of *Brahmamuhurta* (K1)	Willingness for *Brahmamuhurta uttana* (A1)	*Brahmamuhurta uttana* (P1)
Dantadhavana (Tooth brushing)	Ideal frequency of *Dantadhavana* (K2)	Willingness to use medicated tooth powder for *Dantadhavana* (A2)	*Dantadhavana* in contraindicated conditions (P2)
Anjana (Application of collyrium)	Ideal method of *Anjana karma* (K3)	Opinion on Presence of gender bias in the usage of *Anjana* (A3)	*Anjana karma* in contraindicated conditions (P3)
Nasya (Nasal instillation)	Drug Usable for Daily *Nasya* (K4)	Opinion on need of Awareness programmes & Modifications for popularising *Nasya* (A4)	*Nasya* with *Aṇutaila* (P4)
Gandusha (Oil pulling)	Procedure of *Gandusha (K5)*	Belief in the textually written health benefits of *Gandusha* (A5)	Gandusha using indicated dravyas (P5)
Abhyanga (Oil massage)	Mandatory areas of *Abhyanga* in body (K6)	Influence of family on *Abhyanga* in comparison with that of Academics(A6)	*Abhyanga* in mandatory areas of body (P6)
Vyayama (Physical Exercise)	*Lakshanas* of *Ardhashakti Vyayama(K7)*	Opinion on lack of proper physical education facility / faculty in *Ayurveda* colleges(A7)	*Vyayama* by considering *Ritu*, *Sarira* and *Ahara* (P7)
Snana (Bath)	Difference in temperature of *Snanajala* using on different parts of body (K8)	Opinion on more Influence of advertisement & cost in selection of materials for *Snana* in comparison with that of Academics(A8)	*Snana* in contraindicated conditions (P8)
Ahara (Food intake)	Ideal time for food intake(K9)	Willingness for ensuring regular timing & proper digestive power before food intake(A9)	Food intake without mindfulness(P9)
Nidra (Sleep)	Ideal time for sleep (K10)	Willingness to avoid late night sleep (A10)	Sleep After 11 PM (Late night sleep) (P10)

Doc.S1- **KAP QUESTIONNAIRE**

TITLE : KNOWLEDGE, ATTITUDE AND PRACTICE OF SELECTED *DINACARYĀ* PRACTICES AMONG BAMS STUDENTS IN KERALA - A CROSS SECTIONAL STUDY

KNOWLEDGE

K1. What is the time mentioned as *Brāhmamuhūrta* in ayurvedic texts?

a) 1 *muhūrta* before sunrise
b) 2 *muhūrta* before sunrise
c) 3 *muhūrta* before sunrise
d) 4 *muhūrta* before sunrise
e) Don't Know

K2. How often one should perform *Dantadhāvana* in a day?

a) 1 Time/ day
b) 2 Times/ day
c) 4 Times/ day
d) Not needed daily
e) Don't Know

K3. Among the following, which is the ideal method of *Anjana* application?

a) From medial to lateral canthus through waterline behind lower eyelid
b) From lateral to medial canthus through waterline behind lower eyelid
c) Over the lower eyelid below the lower eye lashes
d) Over the upper eyelid below the upper eye lashes
e) Don't Know

K4. Which among the following drug can be daily used for *Nasya*?

a) Ksheerabala Taila
b) Sarshapa Taila
c) Aṇutaila
d) Bala Taila
e) Don’t Know

K5. What is the procedure of *Gandūṣa*?

a) Gargling the liquid in the mouth
b) Holding the liquid in the mouth without movement
c) Holding and gargling the liquid in the mouth
d) Holding the *kalka* in the mouth
e) Don’t Know

K6.Which are the compulsory areas of human body where daily *Abhyanga* is - recommended?

a) Hastha, Pada, Śira
b) Hastha, Śira, Śravana
c) Pada, Śira, Śravana
d) Hastha, Śira, Nasa
e) Don’t Know

K7. Identify the wrong *lakshana* about *Ardhashakti Vyāyāma*

a) Vayu Situated in *Hridaya* reaches *Vaktra*
b) Feel to Sleep
c) Mouth feels dry
d) Forehead, Nse, Joints and Under arms starts to Sweat
e) Don’t Know

K8. Select the correct use of “*Snāna jala*” in body

a) *Seetajala* in Adhakaya and *Ushnajala* in *Urdhakaya*
b) *Ushnajala* in Adhakaya and *Seetajala* in *Urdhakaya*

c) *Ushnajala* in both *Urdhakaya* and *Adhakaya*
d) *Seetajala* in both *Urdhakaya* and *Adhakaya*
e) Don’t Know

K9. Pick out the healthy food habit from the following

a) Consume food within 3 hours of previous food intake
b) Fasting for more than 6 hours after previous food intake
c) Food intake without *Udgara sudhi* and feeling of hunger
d) Food intake in-between 3-6 hours after previous food intake
e) Don’t Know

K10. Identify the healthy sleeping habit for an adult person from the following

a) Sleeping after 11P.M
b) Sleeping in daytime
c) Sleeping for 6 hours
d) Sleeping immediately after eating
e) Don’t Know

ATTITUDE

Tick your opinion on the following statements regarding different *dinacaryā* practices

**Qn. No:**	**STATEMENTS**	**Strongly Agree**	**Agree**	**Disagree**	**Strongly Disagree**	**Not Sure**
A1	Everyone should wakeup daily at *Brāhmamuhūrta*					
A2	Everyone should use medicated tooth powder having *Kashaya/ Katu/ Tiktha* taste for daily brushing					

Contd…..

A3	There exist a gender bias in the usage of *Anjana* (Males are more reluctant to use *Anjana* compared to females)					
A4	Proper public awareness programmes and convenient modifications are necessary to make *Nasya* more socially acceptable					
A5	Daily practice of *Gandūṣa* have positive impact on health as mentioned in Ayurvedic textbooks					
A6	In development of habit of *Abhyanga*, the influence of family members is more compared to that of teachers and textbooks					
A7	There is lack of proper training facility/ faculty in *Āyurvēda* colleges for daily exercise of students					
A8	In the selection of materials for *Snāna,* the influence of advertisements and cost of materials are more than that of teachers and textbooks					
A9	Everyone should ensure regular timing and proper digestive power before food intake					
A10	Everyone should sleep before 11PM					

PRACTICE

How often you do the following activities in your daily life? Put a tick mark on suitable column.

**Qn. No:**	**ACTIVITIES**	**Always** (7 Days/ Week)	**Frequently** (5-6 Days / Week)	**Occasionally** (3-4 Days / Week)	**Rarely** (1-2 Days / Week)	**Never** (0 Days /Week)
P1	Wakeup at *Brāhmamuhūrta* (96 minutes before sunrise)					
P2	Tooth brushing even when you have indigestion / Cough / Dyspnoea / Fever					
P3	*Anjana* application after head bath / Food intake / Night awakening					
P4	*Nasya* using *Aṇutaila / Tilataila*					
P5	*Gandūṣa* using *Tilataila / Ushnodaka*					
P6	*Abhyanga* on Head, Ear and Foot					
P7	*Vyāyāma* by considering *Ritu, Sarira bala* and *Āhāra*					
P8	Immediate bath after food intake / after coming from hot outdoor areas / at noon time					
P9	Using Mobile / T.V/ Newspaper/ Other audio-visual devices , during food intake					
P10	Sleep after 11PM					

Doc.S2 - **KAP QUESTIONNAIRE - SCORING SHEET**

KNOWLEDGE

K1. What is the time mentioned as brahma muhurtha in ayurvedic texts?

(a) 1 Muhurtha before sunrise ………………………………………………… **-2**
(b) 2 Muhurtha before sunrise ………………………………………………… **+2**
(c) 3 Muhurtha before sunrise …………………………………………………. **-2**
(d) 4 Muhurtha before sunrise …………………………………………………. **-2**
(e) Don't Know ………………………………………………………………….. **0**

K2. How often one should perform dantadhavana in a day?

(a) 1 Time/ day ……………………………………………………….....….... **-2**
(b) 2 Times/ day...………………………………………………………….……. **+2**
(c) 4 Times/ day...……………………………………………………….....….... **-2**
(d) Not needed daily..……………………………………………………..…. **-2**
(e) Don't Know ……………………………………………………………………. **0**

K3.Among the following, which is the ideal method of Anjana application?

(a) From medial to lateral canthus through waterline behind lower eyelid ……. **+2**
(b) From lateral to medial canthus through waterline behind lower eyelid…….. **-2**
(c) Over the lower eyelid below the lower eye lashes ………………………….. **-2**
(d) Over the upper eyelid below the upper eye lashes ………………………….. **-2**
(e) Don't Know ………………………………………………………….……. **0**

K4. Which among the following drug can be daily used for Nasya?

(a) Ksheerabala Taila ………………………………………………..………. **-2**
(b) Sarshapa Taila ……………………………………………………………. **-2**
(c) Anu Taila…………………………………………………………......…. **+2**
(d) Bala Taila…………………………………………………………………. **-2**
(e) Don't Know………………………………………………………………. **0**

K5. What is the procedure of Gandusha?

(a) Gargling the liquid in the mouth…………………………………………. **-2**
(b) Holding the liquid in the mouth without movement…………………….…. **+2**
(c) Holding and gargling the liquid in the mouth……………………………. **-2**
(d) Holding the kalka in the mouth………………………………………..…. **-2**
(e) Don't Know ……………………………………………………………....…. **0**

K6. Which are the compulsory areas of human body where daily Abhyanga is recommended?

(a) Hastha, Pada, Sira …………………………………………………………..… **-2**
(b) Hastha, Sira, Sravana ……………………………………………………..… **-2**
(c) Pada, Sira, Sravana ………………………………………………………… **+2**
(d) Hastha, Sira, Nasa …………………………………………..………..... **-2**
(e) Don't Know ……………………………………………………………....…. **0**

K7. Identify the wrong lakshana about Ardhashakti Vyayama

(a) Vayu Situated in Hridaya reaches Vaktra..………………………………. **-2**
(b) Feel to Sleep…………………………………………………………………. **+2**
(c) Mouth feels dry……………………………………………………………..... **-2**
(d) Forehead, Nose, Joints and Under arms starts to Sweat…………………..… **-2**
(e) Don't Know ………………………………………..…..……..……….. **0**

K8. Select the correct use of "Snana jala" in body

(a) Seetajala in Adhakaya and Ushnajala in Urdhakaya……………………... **-2**
(b) Ushnajala in Adhakaya and Seetajala in Urdhakaya ………………………. **+2**
(c) Ushnajala in both Urdhakaya and Adhakaya……………………………….. **-2**
(d) Seetajala in both Urdhakaya and Adhakaya ……………………………... **-2**
(e) Don't Know…………………………………………………………………. **0**

K9. Pick out the healthy food habit from the following

(a) Consume food within 3 hours of previous food intake……………………… **-2**
(b) Fasting for more than 6 hours after previous food intake…………………… **-2**

(c) Food intake without Udgara sudhi and feeling of hunger……………..……. **-2**

(d) Food intake in-between 3-6 hours after previous food intake…………..….. **+2**

(e) Don't Know……………………………………………………………....……. **0**

K10. Identify the healthy sleeping habit for an adult person from the following

(a) Sleeping after 11P.M ……………………………………………...….. **-2**

(b) Sleeping in day time ………………………..…………………………..... **-2**

(c) Sleeping for 6 hours.……………………………………………….…….. **+2**

(d) Sleeping immediately after eating………………………………....…... **-2**

(e) Don't Know…………………………………………………..……..…..…. **0**

ATTITUDE

Tick your opinion on the following statements regarding different dinacarya practices

**Qn. No:**	**STATEMENTS**	**Strongly Agree**	**Agree**	**Disagree**	**Strongly Disagree**	**Not Sure**
A1	Everyone should wakeup daily at brahma muhurtha	**+2**	**+1**	**-1**	**-2**	**0**
A2	Everyone should use medicated tooth powder having Kashaya/ Katu/ Tiktha taste for daily brushing	**+2**	**+1**	**-1**	**-2**	**0**
A3	There exist a gender bias in the usage of Anjana (Males are more relectant to use Anjana compared to females)	**-2**	**-1**	**+1**	**+2**	**0**
A4	Proper public awareness programmes and convenient modifications are necessary to make Nasya more socially acceptable	**+2**	**+1**	**-1**	**-2**	**0**

Contd….

Contd….

A5	Daily practice of Gandusha have positive impact on health as mentioned in Ayurvedic textbooks	**+2**	**+1**	**-1**	**-2**	**0**
A6	In development of habit of abhyanga, the influence of family members is more compared to that of teachers and textbooks	**-2**	**-1**	**+1**	**+2**	**0**
A7	There is lack of proper training facility/ faculty in Ayurveda colleges for daily exercise of students	**-2**	**-1**	**+1**	**+2**	**0**
A8	In the selection of materials for Snana, the influence of advertisements and cost of materials are more than that of teachers and textbooks	**-2**	**-1**	**+1**	**+2**	**0**
A9	Everyone should ensure regular timing and proper digestive power before food intake	**+2**	**+1**	**-1**	**-2**	**0**
A10	Everyone should sleep before 11PM	**+2**	**+1**	**-1**	**-2**	**0**

PRACTICE

How often you do the following activities in your daily life ? Put a tick mark on suitable column.

**Qn. No:**	**ACTIVITIES**	**Always** (7 Days / Week)	**Frequently** (5-6 Days / Week)	**Occasionally** (3-4 Days / Week)	**Rarely** (1-2 Days / Week)	**Never** (0 Days / Week)
P1	Wakeup at brahma muhurtha (96 minutes before sunrise)	**+2**	**+1**	**0**	**-1**	**-2**

Contd….

P2	Tooth brushing even when you have indigestion / Cough / Dyspnoea / Fever	**-2**	**-1**	**0**	**+1**	**+2**
P3	Anjana application after head bath / Food intake / Night awakening	**-2**	**-1**	**0**	**+1**	**+2**
P4	Nasya using Anutaila / Tilataila	**+2**	**+1**	**0**	**-1**	**-2**
P5	Gandusha using Tilataila /Ushnodaka	**+2**	**+1**	**0**	**-1**	**-2**
P6	Abhyanga on Head, Ear and Foot	**+2**	**+1**	**0**	**-1**	**-2**
P7	Vyayama by considering Ritu, Sarira bala and Ahara	**+2**	**+1**	**0**	**-1**	**-2**
P8	Immediate bath after food intake / after coming from hot outdoor areas / at noon time	**-2**	**-1**	**0**	**+1**	**+2**
P9	Using Mobile / T.V/ News paper/ Other audio-visual devices , during food intake	**-2**	**-1**	**0**	**+1**	**+2**
P10	Sleep after 11PM	**-2**	**-1**	**0**	**+1**	**+2**

Tab S2- **PREVALENCE OF PERSONAL / DEMOGRAPHIC DATA**

PERSONAL / DEMOGRAPHIC VARIABLE	DOMAINS IN EACH VARIABLE	FREQUENCY(N)	PREVALENCE
**GENDER**	MALE	55	24.5%
	FEMALE	161	74.5%
**STRATA OF COLLEGE**	Government /Aided college	62	27.7%
	Private college	154	72.3%
**YEAR OF STUDY**	1st and 2nd Year	113	52.3%
	3rd year & above	103	47.7%
**RESIDENCE**	Hostel	169	78.2%
	Home	12	5.6%
	Rent room	35	16.2%
**AYURVEDA FAMILY BACK-GROUND**	With ayurveda family back-ground	39	18.1%
	With out ayurveda family back-ground	177	81.9%
**PRAKRUTI**	Vata	8	3.7%
	Pitta	8	3.7%
	Kapha	6	2.8%
	Vata-Pitta	71	32.9%
	Vata-Kapha	64	29.6%
	Pitta-Kapha	58	26.9%
	Sama	1	0.5%
**SATTVA**	Pravara	28	13%
	Madhyama	171	79.2%
	Avara	17	7.9%
**Socio Economic Status**	APL	177	81.9%
	BPL	39	18.1%

**Diet Type**	Mixed	195	90.3%
	Vegetarian	21	9.7%
**Diet Regularity**	Regular	182	84.3%
	Irregular	34	15.7%
**Bowel Type**	Free	188	87%
	Constipated	28	13%
**Bowel Frequency**	1Time/Day	125	57.9%
	2 Times /Day	86	39.8%
	>2 Times /Day	5	2.3%
**Exercise**	Mild / No Exercise	152	70.4%
	Moderate Exercise	60	27.8%
	Heavy Exercise	4	1.9%
**Sleep type**	Sound	175	81%
	Disturbed	41	19%
**Day sleep duration**	No Day Sleep	177	81.9%
	Day Sleep $\geq$1 hour/day	39	18.1%
**Night sleep duration**	Less than 7 hours/ Day	155	53.2%
	7-9 hours / Day	99	45.8%
	More than 9 hours / Day	2	0.9%
**Body Mass Index (BMI) (Kg/m^2)**	Severely Underweight (<16)	7	3.2%
	Very Underweight (16.0-16.9)	9	4.2%
	Underweight (17.0-18.4)	24	11.1%
	Normal (18.5-24.9)	151	69.9%
	Overweight (30.0-34.9)	22	10.2%
	Obese Class 1 (30.0-34.9)	2	0.9%
	Obese Class 2 (35.0-39.9)	1	0.5%
**Allergy**	Present	75	34.7%
	Absent	141	65.3%
